# In Situ Fabrication of Complex Hollow Nickel Microstructures via Filament-Guided Electrolyte–Column Electrodeposition

**DOI:** 10.3390/mi17091011

**Published:** 2026-08-27

**Authors:** Wei Wang, Taiyu Li, Yongfeng Li, Linchao An, Yunyan Zhang, Lan Chen

**Affiliations:** 1 School of Mechanical Engineering, Henan Institute of Technology, Xinxiang 453003, China; 2 School of Mechanical and Electrical Engineering, Henan Institute of Science and Technology, Xinxiang 453000, China; 3 School of Intelligent Manufacturing, Henan Institute of Information Science and Technology, Hebi 458030, China

**Keywords:** filament-guided electrodeposition, electrolyte column, complex hollow microstructure, in situ fabrication, numerical simulation, microfabrication

## Abstract

Complex hollow metallic microstructures are essential for microelectromechanical systems (MEMS), lab-on-a-chip microfluidics, and bio-integrated devices, yet their fabrication remains challenging because geometric complexity, microscale precision, and high aspect ratios must be satisfied simultaneously. This study proposes filament-guided electrolyte–column electrodeposition (FG-ECD), which couples a removable filament template with a nozzle-confined electrolyte column to define internal channels in situ during localized metal growth, thereby avoiding the collapse risks associated with conventional template removal routes. A two-dimensional axisymmetric multiphysics model reveals that the embedded filament reorganizes the electrolyte into a stable annular flow and shifts the cathodic current density maximum from the substrate toward the advancing dome front, establishing a self-consistent, quasi-stable localized reaction zone, while a parametric sweep shows that the total current scales the current density magnitude without altering its spatial profile. Experiments demonstrate that a current of 3.6 mA produces smooth dome front growth at approximately 20 μm/min, whereas 5.5 mA triggers sustained hydrogen evolution and a transition to cellular deposition. Under optimized conditions, straight, 540° spiral, and R-shaped dual-channel hollow nickel microstructures were fabricated with continuous, collapse-free internal channels of 50 ± 5 μm, aspect ratios exceeding 10:1, and dimensional accuracy within ±35 μm. FG-ECD provides a low-temperature processing route for complex hollow metallic architectures and offers process regulation principles based on co-regulation of the flow field and current density for electrochemical microfabrication.

## 1. Introduction

Complex hollow metallic microstructures are increasingly required in high-performance microsystems, including microelectromechanical systems (MEMS), lab-on-a-chip microfluidic platforms [1], and implantable bio-integrated sensors [2,3]. Their internal channels and external geometries directly determine fluid transport, structural stability, and device-level functionality. For example, spiral hollow microchannels can enhance mixing in microreactors [4] and heat transfer performance in compact thermal management systems [5], whereas irregular hollow conduits are useful for matching tissue-specific geometries in bio-integrated devices and drug delivery systems [6,7]. The manufacture of such components therefore requires not only microscale dimensional accuracy, but also metallic walls with no visible defects, continuous internal channels with no observed collapse or blockage, and stable high-aspect-ratio growth [8,9].

Metal additive manufacturing has enabled design freedom and monolithic integration for macroscale metallic components [10,11]. However, when the target features decrease to the micro- and mesoscale, melt-based routes such as laser powder bed fusion, directed energy deposition, and binder jetting face critical limitations in spatial resolution, surface quality, residual stress and inner-channel control [12,13,14]. Rough surfaces, lack-of-fusion defects, porosity, and microcracking are particularly detrimental for hollow microchannels because they reduce fluid tightness, corrosion resistance, and structural durability [15,16]. Post-processing can mitigate some defects, but it is difficult to apply uniformly inside narrow, curved, or high-aspect-ratio channels.

Template-assisted microfabrication and microelectroforming provide high surface fidelity but remain poorly suited to complex high-aspect-ratio hollow components. Photoresist or metallic sacrificial templates are commonly used to define hollow cavities; however, template removal from long or curved inner channels can induce structural collapse, stress concentration, and surface damage [17,18]. Two-photon polymerization followed by metallization can create complex polymer templates, but the multistep process introduces precision loss, interface adhesion problems, and nonuniform metal coverage on internal surfaces [19]. Laser microcladding offers direct metallic deposition, yet its feature resolution is generally too coarse for microscale hollow channels with fine internal geometries [20].

Electrochemical additive manufacturing and localized electrodeposition are attractive low-temperature routes for metallic microfabrication because material formation is governed by ion transport and electrochemical reduction rather than melting. Localized electrochemical deposition and meniscus-assisted metal printing have produced microtubes, nanowalls, lattices, and helical metallic structures [21,22,23]. Electrolyte column localized electrochemical deposition has further enabled curved nickel micropillars with high dimensional repeatability and smooth surfaces [24]. Nevertheless, most existing approaches remain solid feature writing processes. They lack a built-in mechanism that simultaneously defines, supports, and preserves arbitrary hollow inner channels during continuous metal growth, which restricts their use for complex hollow metallic microstructures [25,26].

To address this processing challenge, this study proposes filament-guided electrolyte–column electrodeposition (FG-ECD). The method integrates a removable filament template, electrolyte–column confinement, and flow field/electric field coupling to achieve localized nickel growth around a predefined inner channel. The objective is to clarify how the embedded filament regulates electrolyte transport and current density distribution, and to demonstrate the fabrication of straight, spiral, and R-shaped hollow nickel microstructures. The work is positioned within the processing–structure–performance framework of materials research: process parameters regulate local current density and deposition morphology, which in turn determine surface integrity, channel fidelity, and structural accuracy.

## 2. Materials and Methods

### 2.1. Principle of FG-ECD

**Figure 1:**
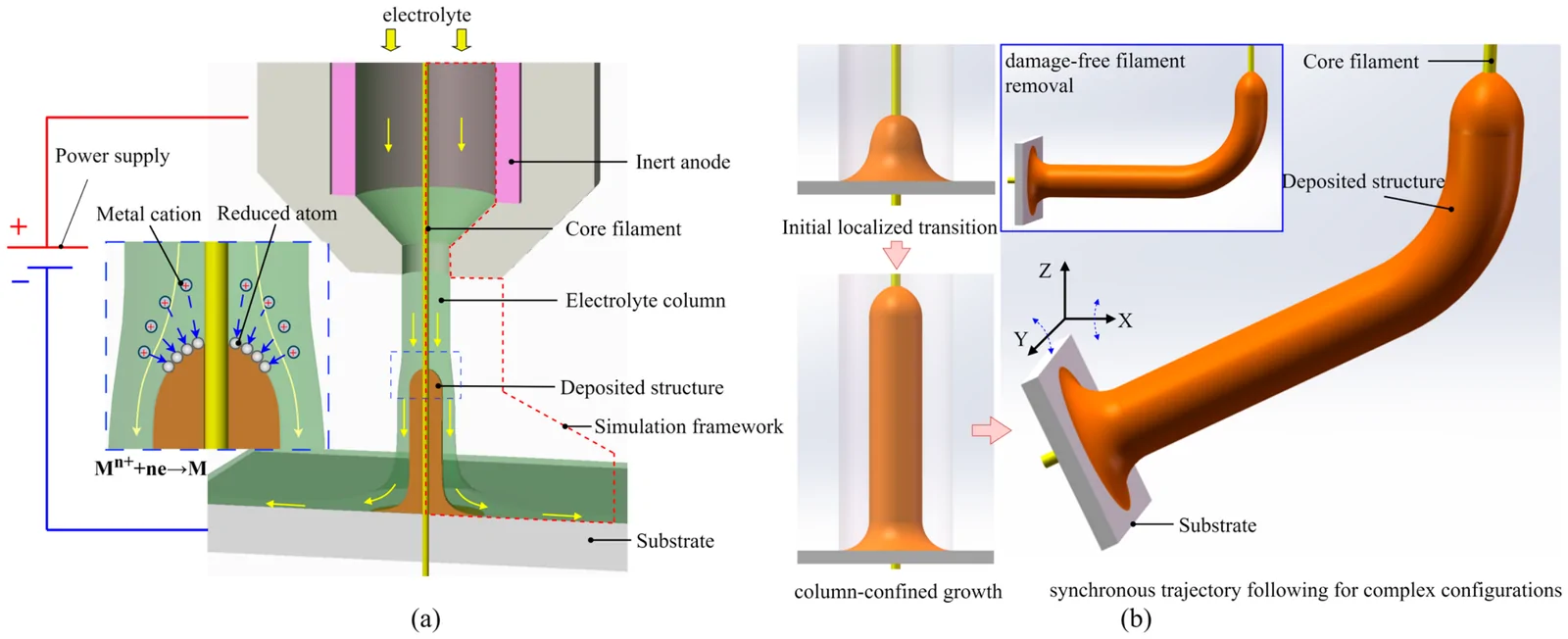
Schematic illustration of FG-ECD for hollow metallic microstructures. (**a**) FG-ECD system and localized electrochemical reaction zone; (**b**) in situ fabrication process for complex hollow metallic microstructures. In (**a**), the blue dashed box denotes the magnified localized electrochemical reaction zone, and the red dashed boxes indicate the region adopted as the 2D axisymmetric simulation framework.

Figure 1 illustrates the FG-ECD concept for fabricating hollow metallic microstructures. The process relies on three coupled functions. First, the filament acts as a removable internal template that defines the inner-channel geometry during electrodeposition. Second, the nozzle-bounded electrolyte column confines the reaction region to a microscale volume and suppresses uncontrolled lateral deposition on non-target substrate areas. Third, the embedded filament reorganizes electrolyte transport into an annular flow field, which improves ion supply around the filament and supports circumferentially uniform metal deposition.

The fabrication sequence consists of localized nucleation on the cathodic substrate, dome front growth around the filament, synchronous trajectory following for curved or branched geometries, and final mechanical filament removal. During growth, the deposited nickel shell develops around the filament instead of being created by post-deposition drilling or sacrificial template dissolution. This in situ channel definition mechanism is the essential difference between FG-ECD and conventional micro-electroforming routes, in which the inner channel is often vulnerable to collapse or damage during template extraction.

### 2.2. Experimental Setup and Fabrication Procedures

A customized FG-ECD system was built for filament positioning, localized electrodeposition, and filament removal. Nylon 6/6 filaments (50 and 100 μm in diameter, 75 MPa tensile strength) were chosen as removable templates for their chemical stability and ease of extraction. They were ultrasonically cleaned in acetone and deionized water for 10 min each, then coaxially aligned with the electrolyte column between the nozzle and cathodic substrate.

The system comprised a platinum tube anode, an acrylic nozzle (540 μm outlet), a four-axis motion stage, and cathodic substrates (0.1 mm bronze sheets or 2 mm copper tubes) (Figure S1). The optimized electrolyte contained 500 g/L nickel sulfamate, 20 g/L nickel chloride, and 30 g/L boric acid, with a pH of 4.2 ± 0.1 and a temperature of 50 ± 1 °C. Deposition current was screened from 1 to 10 mA; 3.6 mA was selected for optimal growth, while 5.5 mA served as a high-current condition to trigger unstable cellular deposition. Electrolyte flow was set at 50 mL/min. For spiral and R-shaped parts, the motion stage drove the filament along the programmed path while the nozzle followed to maintain a stable reaction zone. During fabrication, nozzle motion was synchronized with the motion stage via open-loop feedforward control, with translation speed calibrated to the deposition rate to maintain a constant nozzle-to-cathode distance. To mitigate cumulative positional drift between the growth front and programmed nozzle position, the nozzle-to-deposit distance was periodically verified via side-view optical monitoring, and long structures were fabricated in segments with intermediate realignment.

After deposition, the filament was mechanically removed at room temperature with a tensile force below 10 N. The nylon filament underwent axial elongation and cross-sectional reduction during pulling, which facilitated separation from the inner wall. Low interfacial adhesion allowed clean extraction without damaging the hollow structure (Video S4).

### 2.3. Numerical Simulation Model

A 2D axisymmetric finite element model was developed to study the coupled flow and electric fields during FG-ECD (Material S1). The geometry (Figure S2) matched the experimental setup: nozzle outlet of 540 μm, filament diameter of 100 μm, and an anode–cathode gap of 2 mm. Due to the assumption of rotational symmetry, the model is strictly applicable to straight vertical growth and cannot resolve asymmetric flows or curvature-induced field distortions occurring during complex toolpath execution. Consequently, it serves as a baseline approximation providing qualitative scaling trends for curved geometries, while the fabrication of such structures is validated experimentally in Section 3.4.

Boundary conditions (Table S1) followed experimental parameters: inlet flow rate, outlet at the electrolyte–air interface, constant current at the anode, and current potential at the cathode/deposition surface. A parametric current sweep from 0.002 to 0.005 A was used to quantify how current density scaled with the total current. The simulated trends informed the interpretation of experimental results, explaining the smooth-to-cellular transition. All current density values reported in this work are simulated local values for mechanistic insight; current optimization was based on experimental morphology and growth rate. Mesh refinement was applied near the filament and deposition surface to resolve steep gradients, with a convergence residual < 10^−3^. The simulation is interpreted as a baseline current density scaling model rather than a direct two-phase instability model at the cathode/deposition surface; two-phase bubble effects at high current were discussed qualitatively based on in situ observations.

### 2.4. Characterization Methods

Hollow microstructure morphology was observed with an industrial camera (Microvision EM200C, Microvision, Xi’an, China) equipped with a telecentric lens and an Olympus BX53 optical microscope (Olympus Corporation, Tokyo, Japan). Surface and local microstructure were examined by scanning electron microscopy (SEM; TESCAN Vega 3, TESCAN, Brno, Czech Republic, and Zeiss Sigma 300, Carl Zeiss AG, Oberkochen, Germany), with elemental composition determined via energy-dispersive X-ray spectroscopy (EDS). Computed tomography (micro-CT; Royma RMCT4000, Royma Tech (Suzhou) Precision Co., Ltd., Suzhou, China) assessed porosity and internal channel continuity. The micro-CT scans were acquired at a voxel size of 3 μm, which sets the detection limit for internal voids; possible sub-voxel defects were assessed by SEM cross-sections. The inner-wall roughness was characterized using a 3D laser scanning microscope (OLS5100, Olympus Corporation, Tokyo, Japan). To characterize the local mechanical properties across the microtube wall’s cross-section, depth-sensing nanoindentation was conducted using a nanoindenter (KLA Corporation, Milpitas, CA, USA) equipped with a Berkovich diamond indenter, operated in Continuous Stiffness Measurement (CSM) mode.

## 3. Results and Discussion

### 3.1. Flow Characteristics of the Electrolyte Column with an Embedded Filament

**Figure 2:**
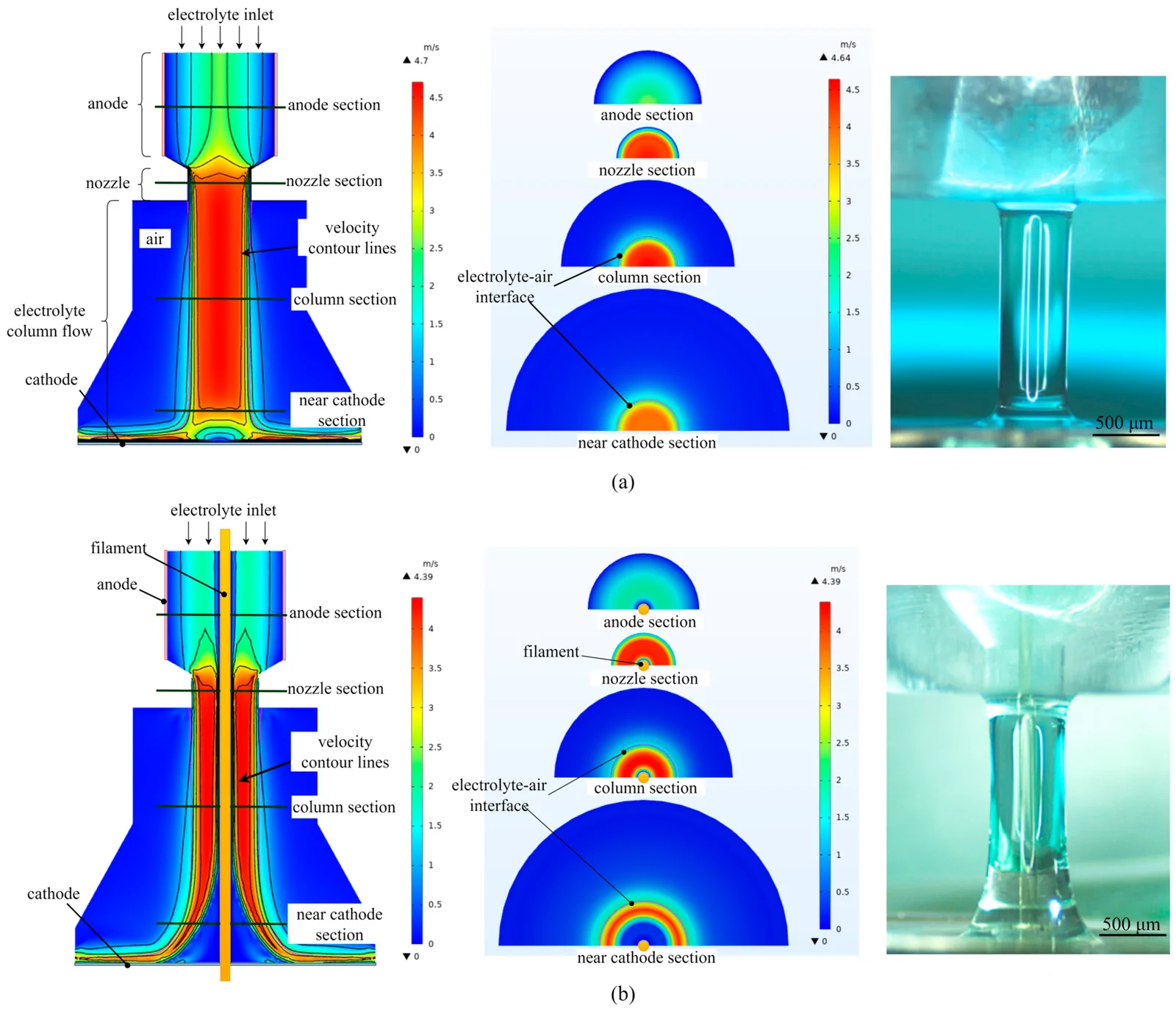
Simulated flow characteristics of the electrolyte column: (**a**) without embedded filament; (**b**) with embedded filament, showing stable annular flow.

The effect of the embedded filament on electrolyte column transport was investigated by numerical simulation, as shown in Figure 2. Both the filament-free and filament-embedded configurations maintained a stable columnar electrolyte flow under the selected nozzle and flow-rate conditions. This is consistent with the interpretation that the nozzle geometry and electrolyte flow rate are sufficient to prevent excessive lateral spreading of the electrolyte column. However, the internal velocity distribution differed substantially between the two configurations. Without the filament, the maximum velocity was concentrated near the central axis and decreased toward the column boundary. After the filament was introduced, it acted as a flow barrier and redistributed the electrolyte into the annular gap between the filament surface and the nozzle wall. The cross-sectional velocity profiles indicated that this annular flow remained stable along the vertical direction of the column, which is beneficial for reducing radial ion concentration gradients during electrodeposition.

The near-cathode flow further illustrates the localization effect of the filament. In the filament-free case, the electrolyte turned sharply at the substrate and spread laterally, which enlarged the region where metal ions could be transported to the cathode. In the filament-embedded case, the annular flow created a larger near-substrate stagnant region and limited the effective reaction zone around the filament. Although the initial cathodic deformation could be influenced by local flow turning at the column base, the upper and middle parts of the electrolyte column remained governed by the nozzle and filament geometry. This stable annular transport provides the fluid dynamic basis for localized and circumferentially uniform hollow structure growth in FG-ECD. Given the two-dimensional axisymmetric nature of the simulation, this flow field interpretation serves as a reference mechanism solely for straight vertical growth. During curved toolpath execution, three-dimensional effects such as circumferential flow asymmetry and curvature-induced secondary flows are not resolved; thus, the model is discussed as a limitation rather than a quantitative predictor for complex geometries. Regarding hydrodynamic shear on the filament, the filament is tautly mounted between the nozzle and the substrate and is effectively constrained by rigidification from the growing nickel shell. Continuous optical monitoring reveals no filament vibration or lateral displacement, which is corroborated by the uniform outer diameter of the fabricated structures.

### 3.2. Evolution Law of Localized Electrodeposition

**Figure 3:**
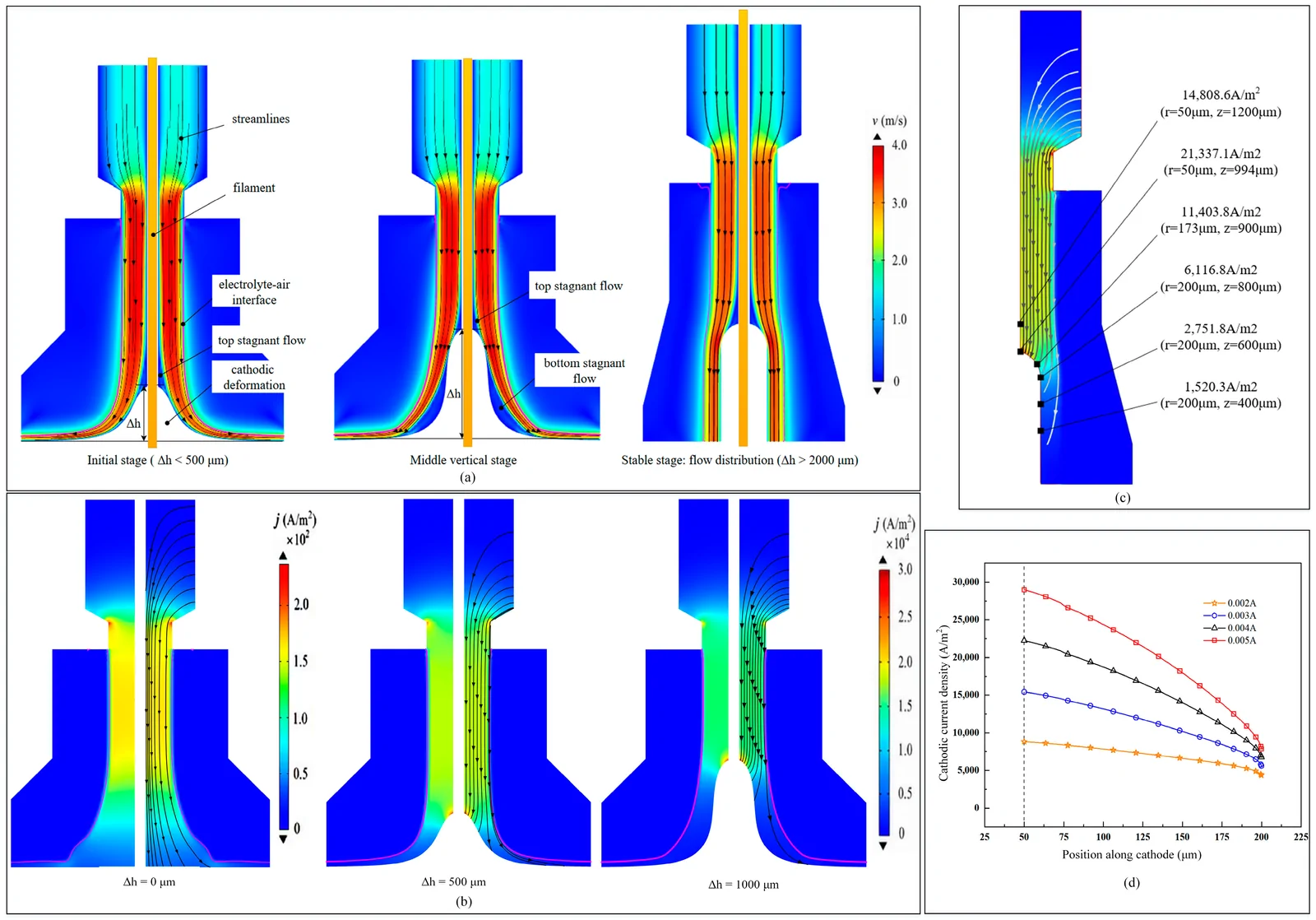
Evolution dynamics of localized electrodeposition in FG-ECD: (**a**) flow field evolution across the initial, middle vertical, and stable growth stages; (**b**) simulated cathodic current density redistribution at deposition height differences (Δh) of 0, 500, and 1000 μm; (**c**) quantified simulated cathodic current density distribution in the stable stage; (**d**) simulated effect of deposition current on cathodic current density distribution. All current density values are simulated local values at the deposition front; they are not nominal average current densities over the entire cathodic surface.

The localized electrodeposition process in FG-ECD is governed by the deposition height difference, Δh, defined as the height difference between the growing cathodic deposit and the initial substrate surface. The coupled flow field, electric field, and morphology results in Figure 3 show that the process can be divided into three stages: initial transition, middle vertical growth, and stable annular film-confined growth. The current density contours and annotated values in Figure 3 are simulated local cathodic current densities; they are used to explain the evolution of the active deposition region rather than to represent direct experimental measurements. These simulated local current densities at the highly confined advancing front, rather than nominal macroscopic averages, exceed those of macro-scale electroplating yet remain consistent with the high current density operation of jet and brush electrodeposition. The high-velocity annular flow continuously renews the diffusion boundary layer at the reaction front, sustaining Ni^2+^ supply and preventing concentration polarization and burnt deposits that would arise at comparable current densities in a static bath.

At the initial transition stage (Δh < 500 μm, Figure 3a), the electrolyte column impinges on the cathodic substrate and turns radially outward, while the embedded filament induces a symmetric annular flow inside the column. Because the deposited nickel is still close to the substrate, the cathodic current density is distributed over a relatively broad near-substrate region. As Δh increases from 0 to 500 μm (Figure 3b), the deposited layer begins to rise around the filament, and the current density maximum gradually moves from the substrate toward the filament-adjacent deposition front. This redistribution indicates that the coupled filament/electrolyte column structure progressively suppresses lateral deposition and converts the active reaction zone from a substrate-dominated region to a filament-guided localized growth region.

During the middle vertical growth stage, the nickel layer grows upward along the filament and forms a preliminary cylindrical shell with a continuous deposition front. The electrolyte column remains confined around the growing shell, while the filament maintains the internal channel geometry. The corresponding current density field becomes increasingly concentrated near the shell front and at the filament–deposit interface. This concentration promotes vertical growth of the advancing front, but the annular flow keeps ion transport sufficiently uniform around the circumference to avoid severe asymmetric deposition. By synchronizing nozzle motion with the growth front and controlling the deposition current, the nozzle-to-cathode distance can be kept approximately constant, producing a columnar hollow shell with a stable outer diameter and a smooth dome-shaped top.

When Δh reaches approximately 1000 μm and further increases toward the stable regime, the cathodic current density distribution becomes more localized along the upper part of the deposit (Figure 3b). At Δh ≥ 2000 μm, the process enters the stable growth stage. The flow pattern changes from direct wrapping of the bare filament to annular film flow surrounding the electrodeposited shell (Figure 3a). At this stage, the current density field becomes quasi-stable. As quantified in Figure 3c, the upper front region exhibits current densities on the order of 10^4^ A/m^2^, while the lower sidewall region has a much lower current density. This axial gradient explains why the dome front remains the dominant growth region and why the previously formed sidewall is not continuously thickened. Thus, the stable stage is controlled by a self-consistent coupling: annular film flow supplies ions to the advancing dome front, and the electric field focuses the electrodeposition reaction in the same region.

The deposition current mainly changes the magnitude of the cathodic current density rather than the spatial distribution profile. As shown in the simulated current sweep in Figure 3d, increasing the total current from 0.002 to 0.005 A raises the current density over the entire active cathodic region, with the peak value increasing nearly proportionally to the applied current. This trend clarifies why a higher experimental current can accelerate growth but also increases the risk of local overpotential at the dome front and the filament–deposit interface. Once the local overpotential becomes excessive, hydrogen evolution is promoted, and electrocrystallization becomes unstable. The current density analysis therefore provides a mechanistic basis for selecting 3.6 mA as the favorable fabrication current identified through parametric comparison and 5.5 mA as an intentionally high-current comparison condition. It is worth noting that electrodeposition propagates along the insulating filament via a triple-phase boundary mechanism: Ni^2+^ reduction occurs exclusively at the conductive metallic dome front, while electrons are transported through the deposited nickel shell. As the shell lengthens, its increasing ohmic resistance shifts the local overpotential toward the dome front, favoring axial over lateral growth—consistent with the current density localization observed in Figure 3c.

### 3.3. Effect of Deposition Current on Deposition Morphology

**Figure 4:**
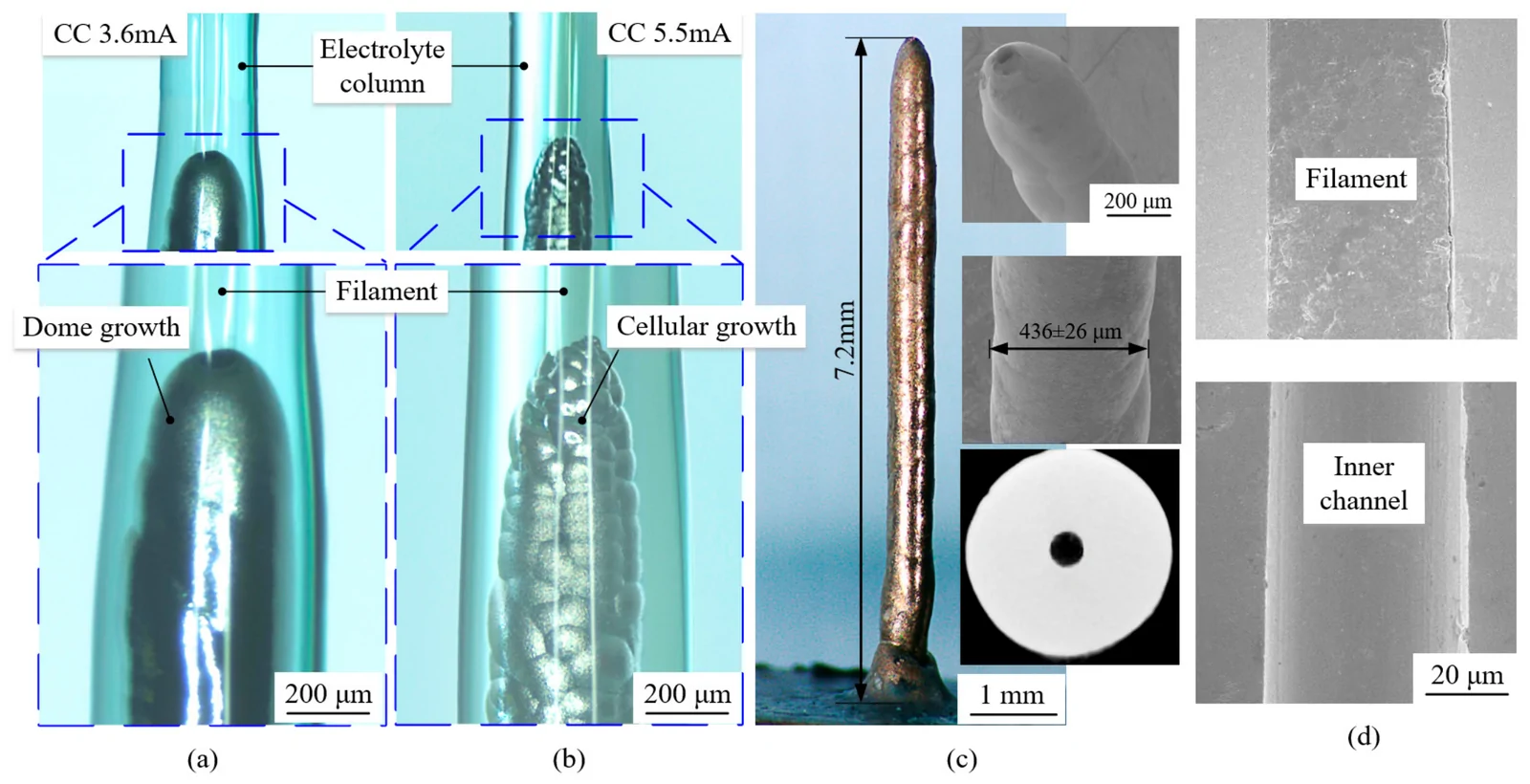
Deposition morphologies and hollow component characterization under different deposition currents: (**a**) smooth dome-shaped growth at 3.6 mA; (**b**) cellular growth with honeycomb-like textures at 5.5 mA; (**c**) straight hollow component with an aspect ratio greater than 10; (**d**) SEM images of the inner-channel morphology.

The current density mechanism identified in Figure 3 is directly reflected in the deposition morphology. Figure 4 compares FG-ECD growth at 3.6 and 5.5 mA. At 3.6 mA (Figure 4a), the deposit maintains a smooth dome-shaped front, and the electrolyte column tightly wraps the filament and growing shell (Video S1). The measured growth rate is approximately 20 μm/min. EDS analysis confirms that the deposited layer is high-purity nickel, with a nickel content exceeding 99.5%. These results indicate that the local current density is high enough to sustain continuous nickel reduction, but not so high as to cause significant hydrogen evolution disturbance.

When the deposition current increases to 5.5 mA (Figure 4b), the morphology changes from smooth dome front growth to cellular growth with rough honeycomb-like textures. The growth rate increases to approximately 50 μm/min, but this apparent efficiency gain is accompanied by surface instability and sustained hydrogen bubble generation (Video S2). The high current magnifies current density hotspots at the dome front and filament–electrolyte interface, causing local ion depletion and increased overpotential. These effects accelerate the hydrogen evolution reaction and disrupt regular nickel electrocrystallization, resulting in cellular deposition. In this FG-ECD system, reducing the current to 3.6 mA effectively suppresses persistent hydrogen evolution and restores smooth, dense deposition; the threshold is process-specific and depends on electrolyte composition, nozzle geometry, flow rate, and filament diameter. This current-dependent morphological evolution aligns with recent studies demonstrating that current density is a critical determinant of the microstructure and performance of electrodeposited nickel-based materials. Because the present numerical model does not explicitly resolve two-phase bubble dynamics, the cellular morphology is described here as being consistent with high-current-induced gas/bubble disturbance and unstable electrocrystallization, rather than as a phenomenon predicted solely by the simulation.

Under the identified favorable current condition, FG-ECD produced straight high-aspect-ratio hollow nickel microcomponents with a height above 7 mm, an outer diameter of 436 ± 26 μm, and an aspect ratio greater than 10 (Figure 4c). Optical and SEM observations show an external surface with high smoothness, and the inner channel is continuous with no visible discontinuities. Cross-sectional characterization of samples fabricated with and without the embedded filament supports the conclusion that the hollow channel is generated by conformal nickel deposition around the filament. After filament removal, the inner-wall profile accurately replicates the filament geometry, and the channel width is controlled at 50 ± 5 μm (Figure 4d).

The combined simulation and experimental results identify 3.6 mA as the favorable operating current for high-quality hollow microstructure fabrication under the present electrolyte, nozzle, and flow conditions. At this current, the annular flow field and the cathodic current density distribution are well matched, enabling efficient growth while avoiding hydrogen evolution-induced defects. In contrast, 5.5 mA produces local current density amplification and a transition to cellular morphology. This processing morphology relationship is important for FG-ECD because the surface quality and channel integrity of the hollow microstructure are directly controlled by the current density field at the moving deposition front. Nanoindentation tests on cross-sections of the electrodeposited nickel microstructure show a microhardness of 5.38–5.73 GPa and an elastic modulus of 155–181 GPa, confirming the mechanical robustness of the deposited nickel shell. These mechanical properties are substantially higher than those of the Nylon 6/6 filament, consistent with the observed damage-free extraction.

### 3.4. Fabrication and Characterization of Complex Hollow Structures

As noted in Section 2.3, the two-dimensional axisymmetric model provides the mechanistic baseline for straight growth; for curved toolpaths, lateral motion of the nozzle relative to the deposit front can induce asymmetric near-filament flow and a mild current density bias toward the inner radius of curvature. The following results therefore serve as the primary experimental demonstration that FG-ECD maintains localized, circumferential deposition under such non-axisymmetric conditions.

#### 3.4.1. Spiral Hollow Structure

**Figure 5:**
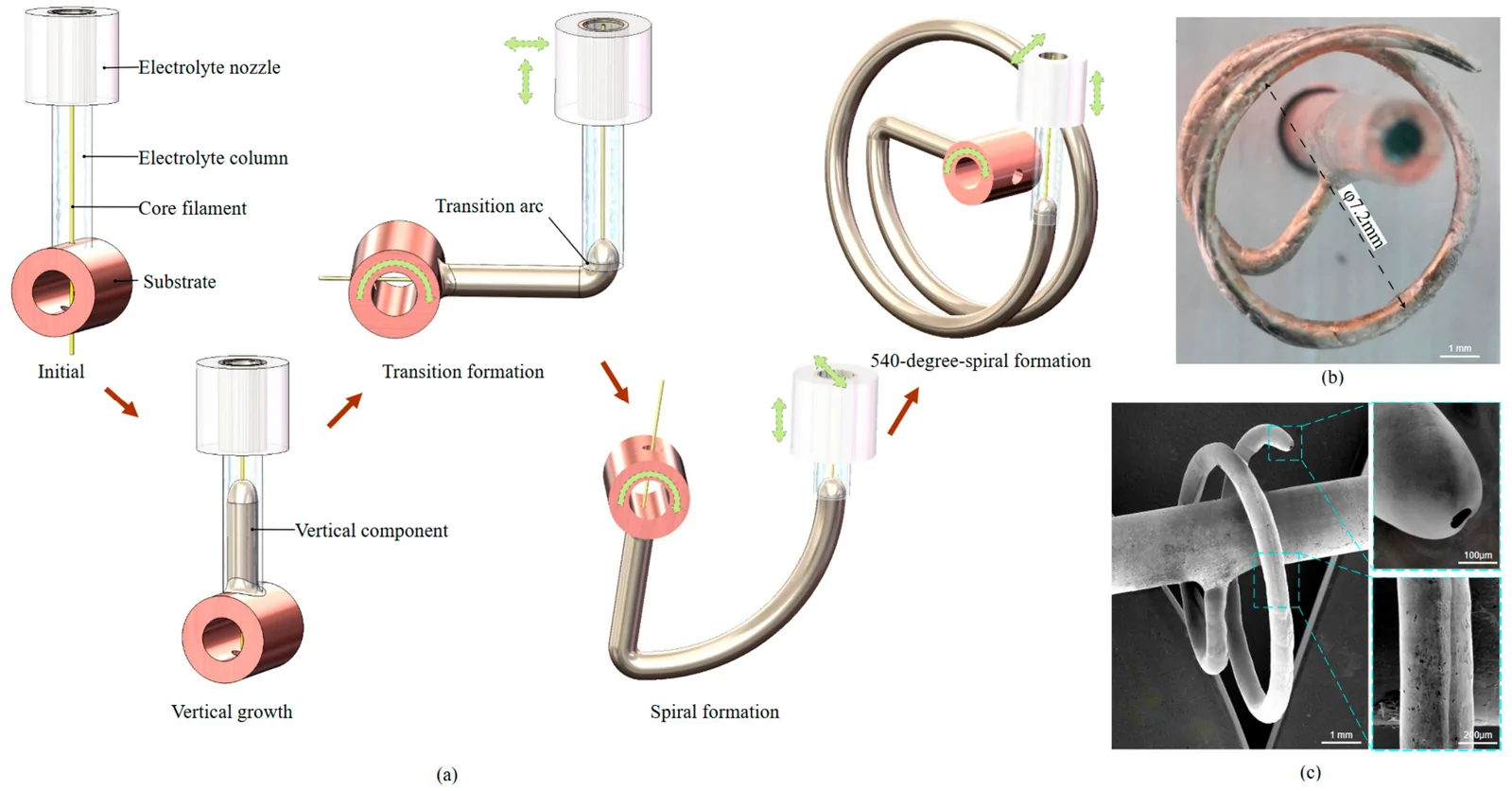
Fabrication and characterization of a 540° spiral hollow structure. (**a**) motion strategy; (**b**) macroscopic morphology; (**c**) SEM image and high-magnification insets. In (**a**), the green arrows indicate the motion directions of the nozzle and substrate, and the dark-red arrows indicate the sequence of fabrication steps.

The capability of FG-ECD for curved hollow microstructure fabrication was evaluated by producing a 540° spiral hollow structure using a 50 μm diameter filament, as shown in Figure 5 and Video S3. The trajectory consisted of vertical growth initiation, transition arc formation, and continuous spiral growth. The fabricated structure achieved a full 1.5-turn configuration with a span of 7.2 mm and an aspect ratio above 10. The macroscopic morphology indicates that the structure remains continuous during curved growth, confirming that the electrolyte column can follow the moving filament while maintaining a localized reaction zone.

SEM observations further confirm the geometric continuity and surface integrity of the spiral component. The spiral tip and middle section exhibit a smooth surface and uniform size, with an average outer diameter of 447 μm and dimensional deviation within ±35 μm. CT analysis (Figure S3) indicates that the internal channel diameter is 50 ± 5 μm, which is consistent with the filament diameter and its dimensional variation. The agreement between the inner-channel geometry and filament profile indicates the high-fidelity microreplication capability of FG-ECD for curved hollow channels. The stable geometry is consistent with the annular flow regulation and localized current density distribution discussed in Section 3.1 and Section 3.2.

#### 3.4.2. R-Shaped Hollow Structure

**Figure 6:**
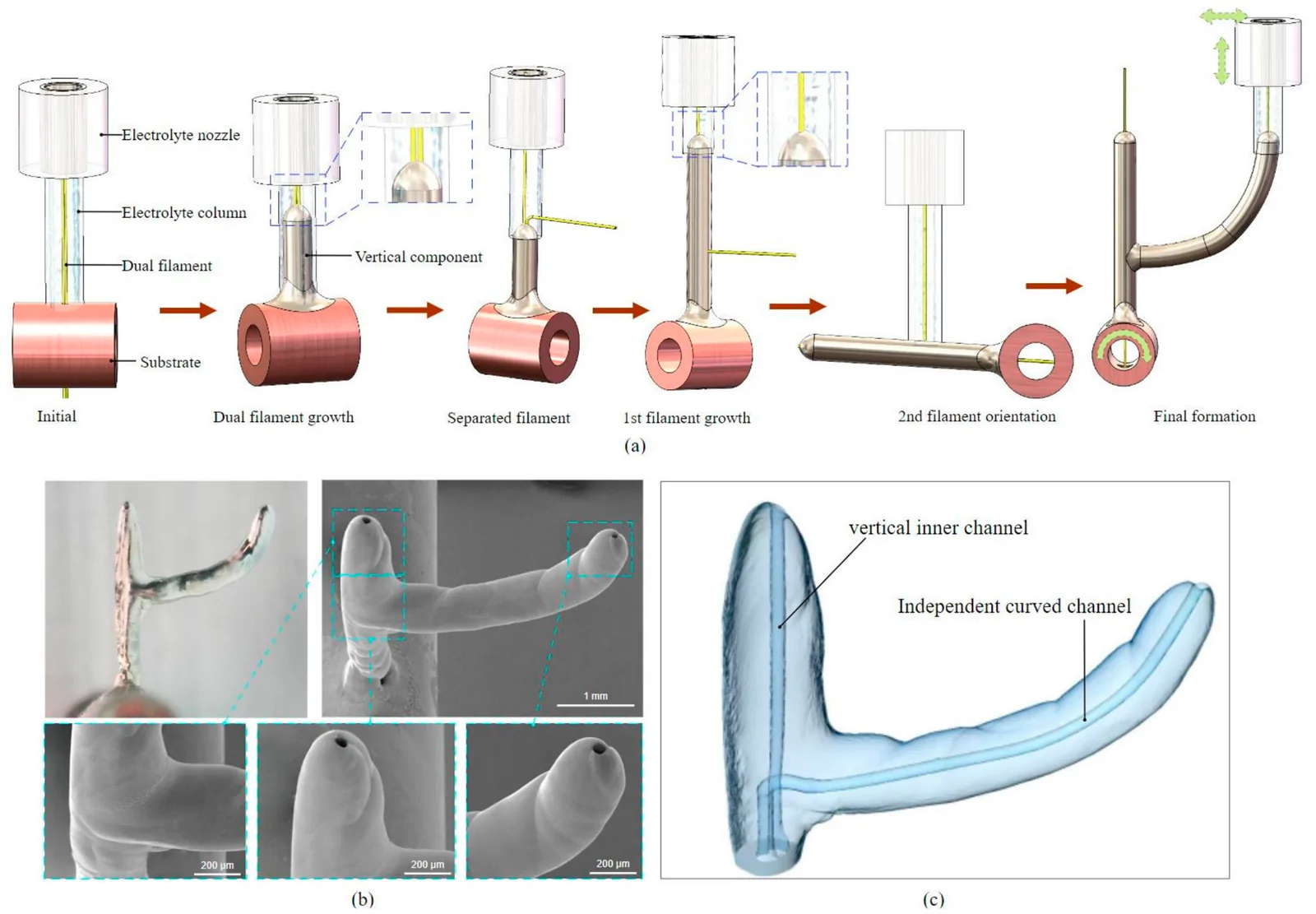
Fabrication and characterization of an R-shaped hollow structure. (**a**) integrated dual-filament fabrication strategy; (**b**) morphology images; (**c**) micro-CT image of the internal channels. In (**a**), the dark-red arrows indicate the sequence of fabrication steps, the green arrows indicate the nozzle motion directions, and the blue dashed-line boxes denote the magnified insets of the nozzle-tip/deposition-front region.

To demonstrate the fabrication of multi-channel irregular hollow geometries, an R-shaped hollow structure was produced using a dual-filament strategy (Figure 6). The process includes dual-filament pre-assembly, first filament-guided electrodeposition, and second filament-guided electrodeposition. Two independent filaments were initially positioned within the electrolyte column, and a vertical hollow segment was first deposited on the cathodic substrate. One filament was then detached from the electrolyte column, while the remaining filament guided the first branch until the designed dimension was reached. For the second branch, the detached filament was repositioned at the nozzle axis, and the relative spatial orientation between the nozzle and the substrate was adjusted to form an isolated secondary internal channel.

The R-shaped component exhibits smooth transitions between the vertical, curved, and horizontal segments (Figure 6b), demonstrating the ability of FG-ECD to maintain localized deposition during irregular trajectory changes. SEM observation shows an external surface with no visible defects across different structural regions. CT imaging directly verifies that the internal channels of the R-shaped structure are continuous and free of collapse or blockage (Video S5). Quantitative measurements show that the relative deviation of the channel diameter is below 5%, confirming that the internal channel remains well defined even under dual-filament and curved-growth conditions. These results suggest that filament guidance and electrolyte column confinement can be extended from simple straight channels to complex, spatially isolated hollow geometries.

### 3.5. Processing Capability and Technical Advantages of FG-ECD

The results demonstrate that FG-ECD provides a localized electrochemical route for hollow nickel microstructures by integrating channel definition with metal growth. Within the processing–structure–performance framework, the annular flow and current density field govern dome front morphology and surface integrity, yielding intact internal channels and high dimensional fidelity. Unlike melt-based additive manufacturing, FG-ECD avoids thermal damage and residual stress under low-temperature conditions. Unlike conventional micro-electroforming, it eliminates post-deposition etching or sacrificial dissolution, as the channel is defined in situ and preserved after filament removal. This coupling of filament guidance and electrolyte confinement enables stable high-aspect-ratio growth and supports programmable motion for curved or branched geometries. Beyond structural fabrication, FG-ECD aligns with MEMS and microfluidic integration: sub-millimeter channels suit lab-on-a-chip thermal management, low-temperature processing protects pre-patterned polymers for post-CMOS and bio-integrated sensors, and single-step R-shaped architectures offer potential for patient-specific drug delivery conduits and neural probes. Three-dimensional transient simulations incorporating toolpath curvature, asymmetric flow, and moving-boundary electrodeposition will be pursued in future work to quantitatively resolve the field distortions in spiral and R-shaped geometries. Future work will also include extended nanoindentation and hardness mapping across different structural regions to further quantify the mechanical robustness of the hollow microstructures for load-bearing microfluidic and MEMS applications.

## 4. Conclusions

This work developed a filament-guided electrolyte column electrodeposition process for in situ fabrication of complex hollow nickel microstructures. By coupling multiphysics modeling with experimental characterization, the underlying transport phenomena and electrochemical kinetics were clarified. The main conclusions are as follows:

(1) FG-ECD integrates filament templating, electrolyte column confinement, and localized electrodeposition, enabling hollow channels to be defined during growth rather than generated by post-deposition machining or sacrificial template dissolution.
(2) The embedded filament reorganizes the electrolyte column into a stable annular flow field. As deposition height increases, the cathodic current density maximum shifts from the substrate to the filament-adjacent dome front and then reaches a quasi-stable distribution during annular film-confined growth.
(3) Deposition current controls the magnitude of cathodic current density and determines deposition morphology. The simulated sweep from 0.002 to 0.005 A clarifies the scaling of current density magnitude, while experiments show that 3.6 mA produces smooth dome front growth and 5.5 mA generates cellular deposition.
(4) Straight, 540° spiral, and R-shaped hollow nickel microstructures were fabricated with continuous, defect-free internal channels and aspect ratios above 10:1.

These findings establish FG-ECD as a viable materials processing method for hollow metallic microstructures and provide process regulation principles based on flow field confinement and current density control.

## Data Availability

The data presented in this study are available on request from the corresponding author.

## Supplementary Materials

The following supporting information can be downloaded at: https://www.mdpi.com/article/10.3390/mi17091011/s1, Figure S1. Photograph of the customized experimental system. Figure S2. Two-dimensional axisymmetric simulation model for FG-ECD. Figure S3. Cross-sectional characterization of the spiral hollow structure via computed tomography (CT) scanning. Material S1: Specific governing equations for multi-physics field coupling. Table S1. Domain and boundary conditions for the numerical simulation of FG-ECD. Video S1. FG-ECD process at 3.6 mA. Video S2. FG-ECD process at 5.5 mA. Video S3. 540° spiral hollow structure. Video S4. Removing the core filament. Video S5. CT of the dual-channel hollow structure.
