# Supplementary material for: In Situ Fabrication of Complex Hollow Nickel Microstructures via Filament-Guided Electrolyte–Column Electrodeposition
Source: Micromachines (Basel). 2026 Aug 27;17(9):1011. doi: 10.3390/mi17091011 (PMC13609239; doi:10.3390/mi17091011)
Supplement: Supplementary file 1 [file micromachines-17-01011-s001.zip › micromachines-4503549-supplementary.pdf]

Supporting Information

This supplementary information provides detailed experimental system characterization, the two-dimensional (2D) axisymmetric numerical simulation model, the specific governing equations for the multi-physics field coupling, and the domain/boundary conditions for the filament-guided electrolyte-column electrodeposition (FG-ECD) simulation, which complement the main text and support the experimental and simulation results presented. Supplementary videos of the electrodeposition process, the fabricated structures, the filament-removal procedure, and the CT reconstruction are also provided.

S1. Supplementary Figures

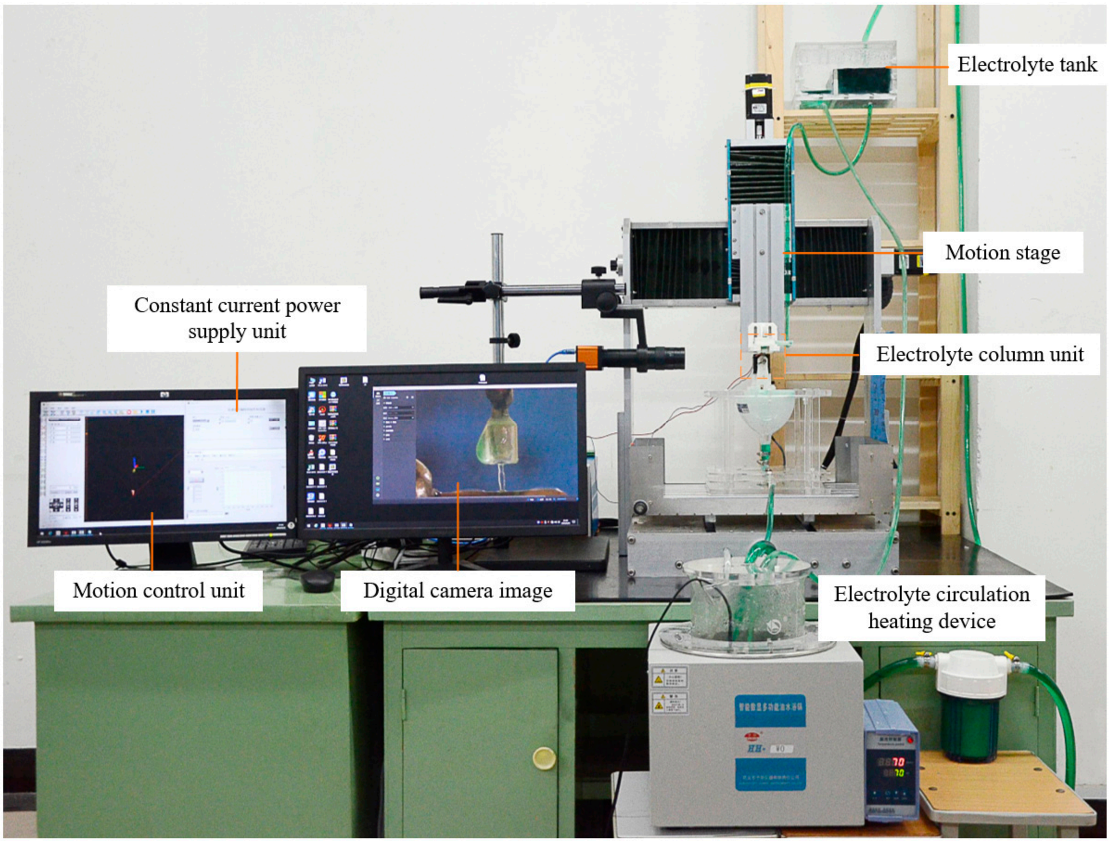

Figure S1. Photograph of the customized experimental system.

The photograph displays the integrated experimental setup for the FG-ECD process, which is composed of six core functional modules: a precision electrolyte supply system (peristaltic pump, electrolyte reservoir, and delivery pipeline) with a controllable flow rate (0–200 mL/min), a platinum tube inert anode (outer diameter 2 mm, inner diameter 1 mm) connected to a DC power supply, a customized acrylic deposition nozzle with a fixed outlet diameter of 540 μm, a four-axis precision motion stage (positioning accuracy ±1 μm) for programmable trajectory control of the filament and nozzle, a filament template assembly unit (for coaxial fixation of Nylon 6/6 filaments with diameters of 50 μm or 100 μm), and a DC constant current power

supply (output range 0–100 mA, precision ±0.1 mA) for regulating electrodeposition current. The cathodic substrate (0.1-mm-thick bronze sheet or 2-mm-diameter copper tube) is fixed on the motion stage workbench, and the entire system is placed on an anti-vibration optical platform to eliminate external interference during microfabrication. The electrolyte column is stably formed between the nozzle outlet and the cathode substrate, and the filament is coaxially embedded in the electrolyte column to realize guided electrodeposition.

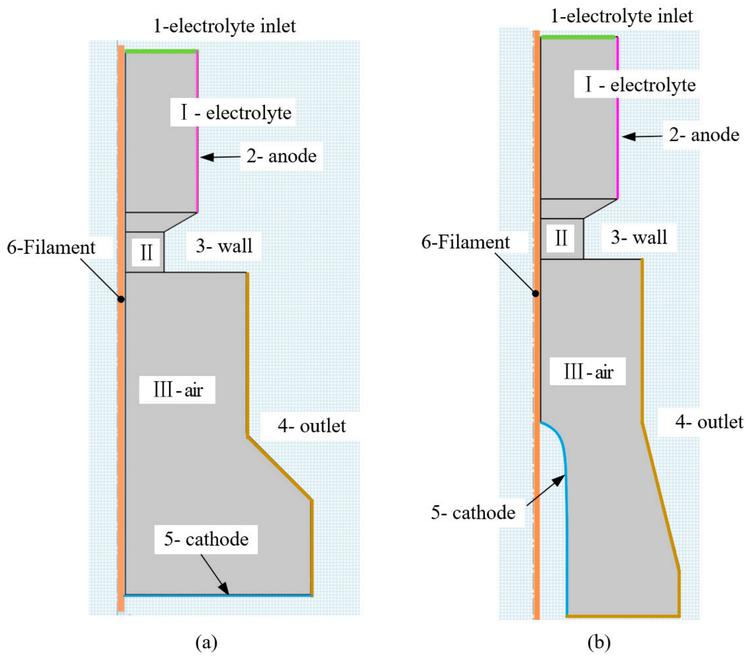

Figure S2. Two-dimensional axisymmetric simulation model for FG-ECD.

The 2D axisymmetric geometric model is reconstructed based on the actual FG-ECD experimental system. The model consists of six key functional regions: platinum tube anode (outer diameter 2 mm, inner diameter 1 mm, length 2 mm), acrylic nozzle (outlet diameter 540 μm, length 5 mm), Nylon 6/6 filament (diameter 100 μm, consistent with the experimental template), electrolyte column (the main reaction region, with an anode-cathode distance of 2 mm), cathodic substrate (bronze sheet, thickness 0.1 mm), and air region (surrounding the electrolyte column to simulate the electrolyte-air interface). All dimensional parameters of the simulation model are strictly consistent with the experimental setup (Section 2.3 in the main text). It is important to note that the 2D axisymmetric simplification inherently assumes rotational symmetry around the central filament axis; therefore, it cannot capture circumferential flow variations, asymmetric ion-transport effects, or the trajectory-curvature-induced field distortions that occur in spiral and R-shaped geometries. The model is most accurate for straight vertical growth and provides qualitative scaling trends for curved structures rather than quantitative local-field predictions. Mesh refinement is performed in the key reaction regions (e.g., the filament surface, electrolyte column near-cathode section, and deposition interface) with a minimum mesh size, while a relatively coarse mesh is adopted in the non-reaction regions to balance simulation accuracy and calculation efficiency. The model is built and solved using the finite element method, coupling the laminar flow module and the electric field module to realize the comprehensive simulation of the fluid-electric multi-physics field in the FG-ECD process.

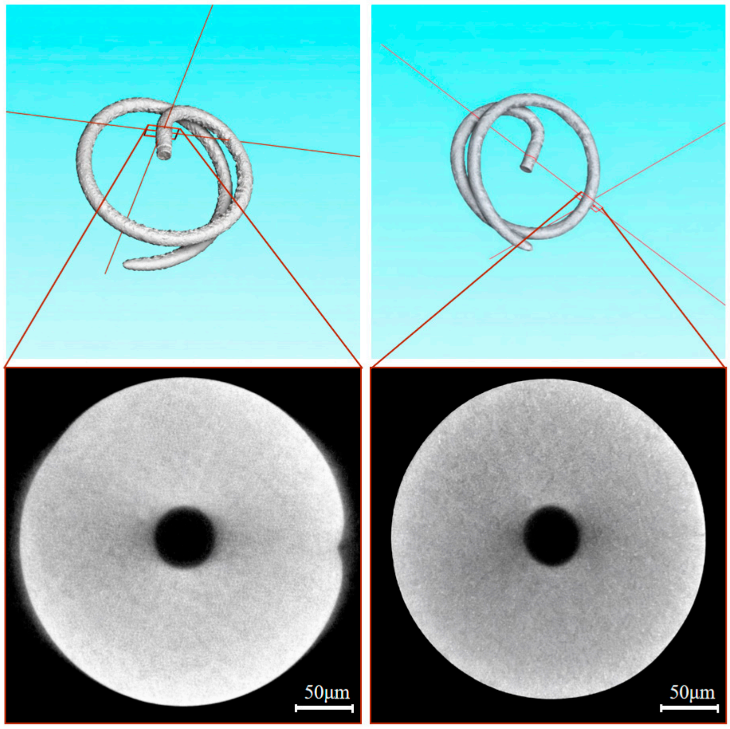

Figure S3. Cross-sectional characterization of the spiral hollow structure via computed tomography (CT) scanning.

Figure S3 presents the cross-sectional analysis of the spiral hollow structure characterized by computed tomography (CT) scanning. The top panels display 3D reconstructed renderings of the spiral architecture, while the bottom panels show corresponding cross-sectional slices acquired at two distinct positions along the filament. In each cross-section, the central circular void corresponds to the hollow core of the constituent filament, and the surrounding annular region exhibits a dense microstructure with well-defined inner and outer surfaces. Both cross-sections are confirmed to possess zero porosity, verifying the structural compactness and integrity of the fabricated hollow structure throughout the spiral geometry.

S2. Specific Governing Equations for Multi-Physics Field Coupling (Material S1)

The multi-physics numerical simulation model of FG-ECD couples the laminar flow field (electrolyte flow) and the electric field (electrodeposition reaction), with the electrolyte regarded as an incompressible Newtonian fluid and the electrodeposition process as a steady-state electrochemical reaction. The specific governing equations for each physical field and the coupling relationship are as follows, with all physical quantities defined using international standard units (SI).

S2.1 Governing equations for laminar flow field (electrolyte column)

The laminar flow of the electrolyte column is governed by the continuity equation and the Navier-Stokes equation, which describe the velocity distribution and flow state of the incompressible electrolyte in the FG-ECD system:

$$\rho\left(\frac{\partial u}{\partial t}+u\cdot\nabla u\right)=-\nabla p+\mu\nabla^2 u+F$$

where ρ is electrolyte density (1.3 × 10³ kg/m³), u is the velocity vector of the electrolyte (m/s), p is pressure (Pa), μ is dynamic viscosity (0.001 Pa·s), and F represents surface tension forces at the electrolyte-air interface.

The level-set method tracked interface evolution using the equation:

$$\frac{\partial\varphi}{\partial t}+u\cdot\nabla\varphi=0$$

where φ is the signed distance function distinguishing electrolyte ($\varphi<0.5$) and air ($\varphi>0.5$).

S2.2 Governing equations for electric field (electrochemical deposition)

The electric field in the electrolyte column follows the Maxwell's equations for steady-state conduction, and the electrodeposition reaction on the cathode surface is controlled by the charge conservation law. The key governing equations include the Laplace equation for potential distribution (electrolyte is a conductive medium with no free charge accumulation) and the current density equation (Ohm's law in vector form):

$$\nabla\cdot\sigma\nabla\Phi=0$$

with σ as electrolyte conductivity (10 S/m) and Φ as electric potential (V). Conductivity variations at the gas-liquid interface were incorporated to account for bubble-induced resistivity changes.

The cathode surface is the reaction interface where metal cations are reduced to metallic atoms, following the reaction: $M^{n+}+ne^{-}\rightarrow M$. The local current density at the cathode surface is consistent with the reaction rate, and the boundary condition of constant current is applied to the cathode surface (Section 2.3 in the main text) to match the experimental electrodeposition parameters. The Butler-Volmer equation described electrodeposition reaction kinetics:

$$j=j_0\left(exp\left(\frac{\alpha_a F\eta}{RT}\right)-exp\left(-\frac{\alpha_c F\eta}{RT}\right)\right)$$

where j_0 is exchange current density, α_a/α_c are transfer coefficients, $\eta=\Phi_{cathode}-\Phi_{eq}$ is overpotential, and F, R, T denote Faraday's constant, gas constant, and temperature, respectively.

S2.3 Coupling relationship between flow field and electric field

The flow field and electric field are coupled through the ion transport process in the electrolyte: the annular flow field induced by the filament homogenizes the electrolyte velocity distribution around the filament, which eliminates the radial ion concentration gradient in the electrolyte column and ensures uniform ion transport to the cathode surface. The uniform ion transport further leads to a homogeneous local current density distribution (electric field), which is the core regulatory mechanism of FG-ECD for uniform electrodeposition. In the numerical model, the flow field results (velocity distribution) are used as the input condition for ion transport, and the electric field distribution is solved based on the homogenized ion transport state, realizing the one-way coupling of the two physical fields. The present simulation is treated as a single-phase baseline electrochemical model. It does not explicitly resolve hydrogen-bubble nucleation, bubble expansion, bubble detachment, void-fraction evolution, or gas-liquid boundary displacement; these high-current two-phase effects are discussed qualitatively based on the in situ video evidence.

S3. Domain and Boundary Conditions for FG-ECD Simulation (Table S1)

The boundary conditions of the 2D axisymmetric simulation model are strictly set in accordance with the experimental operating parameters of FG-ECD (Sections 2.2 and 2.3 in the main text), including the physical quantity type, boundary condition type, and specific

values/descriptions for each domain and key interface. The finite element solution adopts a residual error threshold of $<10^{-3}$ as the convergence criterion to ensure the reliability of the simulation results.

Table S1. Domain and boundary conditions for the numerical simulation of FG-ECD.

Domain/Boundary	Physical Quantity	Boundary Condition Type	Mathematical Formulation
Platinum tube anode	Electric potential	Constant current	Uniform normal flux: $n \cdot J = I/A\_anode$, with I = 3.6 or 5.5 mA
Cathodic substrate (deposition surface)	Local current density	Constant, deformed boundary	**Φ = 0 V; normal current-density flux $n \cdot J = j\_local$**
Core filament surface	Velocity	No-slip wall	u = 0 m/s; electrically insulating: $n \cdot J = 0$
Acrylic nozzle wall surface	Velocity	No-slip wall	u = 0 m/s; electrically insulating: $n \cdot J = 0$
Electrolyte-air interface	/	Interface	Level-set: $\varphi < 0.5$ (electrolyte), $\varphi > 0.5$ (air)
Electrolyte inlet (nozzle top)	Velocity	Constant velocity	Uniform axial velocity corresponding to Q = 50 mL/min
Outlet	Pressure	Pressure outlet	$p = p\_atm$ (atmospheric pressure); surface tension neglected
Symmetry axis (model center)	Velocity/potential	Axis symmetry	Radial derivatives: $\partial u/\partial r = 0$, $\partial \Phi/\partial r = 0$

S4. Supplementary Videos

Video S1. FG-ECD process at 3.6 mA. In situ view inside the electrolyte column during deposition at the optimized current. The nickel deposit advances along the filament with a smooth, bullet-shaped dome front; the electrolyte column stably wraps the growing shell, and no bubble disturbance is observed throughout the deposition.

Video S2. FG-ECD process at 5.5 mA. In situ view of deposition at the high-current comparison condition. Rows of hydrogen bubbles continuously nucleate, grow, and detach at the reaction zone, and the deposit develops a dark, rough nodular (cellular) morphology, in sharp contrast to the smooth growth shown in Video S1. The bubble observations in this video serve as qualitative evidence for the two-phase effects discussed in the main text; they are not outputs of the single-phase simulation.

Video S3. 540° spiral hollow structure . The as-fabricated 1.5-turn spiral hollow nickel structure grown on a 2-mm-diameter copper-tube substrate, shown and rotated against a millimeter reference scale. The surface is bright and smooth, with an overall span of approximately 7 mm.

Video S4. Removing the core filament. Close-up of the room-temperature mechanical extraction of the translucent nylon filament from the branch of an R-shaped (bifurcated) hollow nickel component. The hollow architecture remains intact during extraction, demonstrating damage-free core removal.

Video S5. CT of the dual-channel hollow structure. Rotating micro-CT three-dimensional reconstruction of the R-shaped dual-channel component. Cross-sections at both ends reveal two independent internal channels that are continuous along the entire structure, without collapse or blockage.
